# Intercultural adaptation and influencing factors analysis of the Chinese version of the anxiety scale for the older adults in a long-term care population

**DOI:** 10.3389/fpubh.2023.1270284

**Published:** 2023-10-17

**Authors:** Fuzhe Feng, Qing Chen, Chen Zheng, Huijun Zhang

**Affiliations:** Department of Nursing, Jinzhou Medical University, Jinzhou, China

**Keywords:** anxiety, assessment, older adults, factor analysis, linear regression

## Abstract

**Objective:**

The purpose of this study was to translate the Geriatric Anxiety Scale-Long-Term Care into Chinese and to assess its reliability and validity in a long-term care population, as well as to explore factors contributing to anxiety in older adults requiring long-term care.

**Methods:**

The study recruited 399 older adults residents requiring long-term care and used the Brislin double translation-back-translation method to create the initial Chinese version of the Geriatric Anxiety Scale-Long-Term Care. The study used internal consistency and split-half reliability to assess the reliability of the scale, as well as exploratory factor analysis, validation factor analysis, and content validity to assess the validity of the scale. Linear regression was used to analyze the relationship between the independent variables and anxiety levels in the long-term care population.

**Results:**

The Cronbach’s coefficient value of the Chinese version of the Geriatric Anxiety Scale-Long-Term Care was 0.81, and the split-half reliability was 0.80. The results of exploratory factor analysis showed support for a one-dimensional factor structure. The results of the validation factor analysis indicated a good fit for the one-factor model. Gender (*β* = 0.190, 95% CI:0.540 ~ 1.546, *p* < 0.001), self-rated health (*β* = 0.220, 95% CI:0.379 ~ 0.953, *p* < 0.001), life satisfaction (*β* = −0.315, 95% CI: −1.355 ~ −0.734, *p* < 0.001) and participation in activities (*β* = −0.106, 95% CI: −1.122 ~ −0.084, *p* < 0.05) were significant predictors of anxiety levels in the long-term care population.

**Conclusion:**

The Chinese version of the Geriatric Anxiety Scale-Long-Term Care has good reliability and validity in the long-term care population. The Geriatric Anxiety Scale-Long-Term Care is effective in assessing the anxiety level of the Chinese long-term care older adults population and provides an opportunity to detect and observe anxiety disorders in the long-term care population.

## 1. Introduction

The aging of the population is a major concern around the world. By the end of 2021, The population over the age of 65 was 20.56 million, and the population over the age of 60 was 267.36 million, making up 14.2 and 18.9% of the total population in China. The dependence rate for seniors 65 and over in China is 20.8% (1). The issue of old age has grown in importance as the population has aged.

The accelerated pace of aging, the increasing proportion of disabled and semi-disabled individuals, and the rising prevalence of chronic diseases have led to a continuous expansion of the demand for long-term care among the older adults. Moreover, the diseases suffered by older adults individuals are mostly chronic or age-related, with long recovery times and low cure rates, requiring prolonged long-term care. However, due to economic, work, and family pressures, family members are unable to provide the necessary care, leading to the need for long-term care facilities to care for the older adults. Research has shown that nearly 46% of all individuals aged 65 and above require long-term care services at some point, with more than half of them being admitted to long-term care facilities for care (2).

Anxiety has been found to be one of the most prevalent psychiatric disorders among older adults (3). Older people requiring long-term care often have anxiety symptoms and disorders that are exacerbated by admission to an LTC, and for older people, admission to an LTC creates greater mental stress, particularly in the first 4 weeks of admission (4). International epidemiological studies have shown that at least one-third of people living in LTCs will exhibit clinically significant symptoms of anxiety (5). Older people in LTCs are also more prone to anxiety because they are more frail, unfamiliar with their caregivers, most will have various comorbidities, and they are socially disconnected and at risk of losing their autonomy (6–8).

Anxiety in the LTC population can lead to poorer well-being (9), sleep problem (10), disability burden (11), reduced memory and executive functioning (12), and increased caregiver burden (13). Chang et al. (14) analyzed 247 older persons aged 60 years and older with anxiety and found a significant 2.05-fold increase in mortality compared to those without anxiety. Anxiety symptoms complicate caregiving and can significantly increase the workload of caregivers. Anxiety also imposes a heavy social burden, both directly in the form of personal distress and indirectly in the form of a substantial need for medical support to manage anxiety-induced physical symptoms. The underestimate, underdiagnosis, and subsequent undertreatment of this category of illnesses may increase these socioeconomic costs (3).

Anxiety symptoms in older adults residents of long-term care facilities cannot be ignored, and attention must be paid to screening for anxiety symptoms and their risk factors, and treating them promptly. However, because LTC facilities always adopt a model of care that prioritizes the physical requirements of senior citizens, residents’ anxiety symptoms and disorders are also often overlooked and under-treated (15–17). The reason for this situation may be due to a shortage of personnel in long-term care facilities. According to international standards, the ratio of caregivers to disabled older adults individuals should be 1: 3, and China needs at least 14 million caregivers. However, there are currently only 300,000 caregivers, of which only 40,000 have obtained qualifications for older adults care.

With the increasing number of older adults individuals living in long-term care facilities, there is an urgent need for accurate diagnosis and treatment of anxiety in this population. Meanwhile, China is facing a shortage of caregivers, making it particularly important to have a reliable, effective, and efficient tool for identifying anxiety disorders in long-term care facility residents. The Geriatric Anxiety Scale-Long-Term Care is a self-report assessment scale consisting of ten items that are answered directly with a “yes” or “no” format, making it easy for diagnosis. Currently, there is no specific measurement tool for anxiety disorders in older adults individuals requiring long-term care in China. Therefore, the purpose of this study is to introduce and evaluate the reliability and validity of the Geriatric Anxiety Scale-Long-Term Care scale in China and to explore the factors that influence anxiety in this population.

## 2. Methods

### 2.1. Participants

This cross-sectional study was conducted from May 2022 to October 2022 and involved eight long-term care facilities in Nanyang City, Henan Province, China. The researchers traveled to the eight long-term care facilities after receiving relevant training and recruited participants with the assistance of the long-term care facility supervisors. Participants were recruited from the facilities through convenience sampling, and participants included 399 older adults. Inclusion criteria required participants to be older adults greater than or equal to 60 years of age, in need of long-term care and volunteering for this study. Participants were excluded from the study when their perceptions interfered with their ability to fully understand the study and give informed consent. Participants were contacted by the researcher and after permission to participate and informed consent was obtained, participants completed an anonymously translated scale at their residence. In addition, to explore the factors influencing anxiety in the long-term care population, a further 374 data were collected for a follow-up study from November 2022 to March 2023 for this study. It is recommended to have a sample size for exploratory analysis that is 5–10 times the number of variables in the project (18). For regression analysis, a sample size of 20 times the number of predictor variables is suggested (19). In this study, the scale used consists of 10 items and there are 11 predictor variables. Therefore, the sample size of this study meets the requirements.

### 2.2. Translation process

Before starting the study, we had obtained permission and approval from Prof. Segel (2) to develop the Chinese language copyright of the tool. To ensure accuracy, we employed the principle of double back-translation, as recommended by Brislin (20). Initially, two bilingual native Chinese speakers translated the scale into Chinese. The research team then reviewed and discussed any obvious differences between the translated version and the original scale. Next, two English-speaking foreign scholars, who were not familiar with the original scale, back-translated the Chinese version into English. The research team compared and discussed the original scale, the Chineseized first draft, and the back-translated English scale to create a preliminary draft of the Chinese version. Additionally, a psychologist was consulted to make cultural adjustments to the Chinese version, making it more suitable for Chinese reading habits. To assess the comprehensibility of the scale, we selected 20 older adults in need of long-term care to participate in a pre-survey. The participants reported that the scale was well-structured and easy to understand. Overall, we took extensive steps to ensure the accuracy and cultural appropriateness of the Chinese version of the Geriatric Anxiety Scale-Long-Term Care scale.

### 2.3. Instruments

The study questionnaire included demographic information and original scales. Demographic information includes age, gender, marital status, educational attainment, smoking history, drinking history, frequency of interaction with children, self-rated health, life satisfaction, whether officially retired, and activity participation. The Geriatric Anxiety Scale-Long-Term Care (2) is a self-assessment tool that effectively assesses anxiety levels in older adults receiving long-term care. The scale is specifically designed to assess anxiety over the past week and is therefore a valid tool for monitoring changes in anxiety levels over time. The Cronbach’s coefficient for this scale is 0.81.

### 2.4. Ethics consideration

This study protocol was approved by the relevant Ethics Committee of Jinzhou Medical University (Ethics approval number: JZMULL2022095), and the study was conducted in accordance with the ethical guidelines of the Declaration of Helsinki.

### 2.5. Data analysis

This study used Mplus 8.0 and SPSS 25.0 for data analysis. A robust weighted least squares (WLSMV) estimator that was modified for mean and variance was utilized because the data in question was categorical. WLSMV, a trustworthy estimator that does not assume normally distributed variables, is the best choice for modeling categorical data (21).

#### 2.5.1. Reliability analysis

A reliability test was conducted to assess the internal consistency of the Geriatric Anxiety Scale-Long-Term Care scale, including the computation of Kuder–Richardson-20 (KR-20) coefficient, split-half reliability, and corrected item-total correlations. The acceptable value for KR-20 coefficient, which indicates internal consistency, is 0.7 or above (22). The acceptable value for the corrected item-total correlations, which indicates the overall relatedness of the items, is 0.3 or above (22).

#### 2.5.2. Validity analysis

##### 2.5.2.1. Discriminant validity and factors correlation

The Geriatric Anxiety Scale-Long-Term Care Chinese version of the scale was ranked from highest to lowest total score and the relationship between the top 27% (high subgroup) and the bottom 27% (low subgroup) was analyzed to determine whether the translated scale was appropriately differentiated. Correlations between items and the translation scale and changes in Cronbach coefficient values if items were removed were examined to assess whether each item of the translation scale could be preserved.

##### 2.5.2.2. Content validity

In this study, seven experts were invited to assess the content validity of the Chinese version of the Geriatric Anxiety Scale-Long-Term Care by calculating the item content validity index (CVI) and the mean S-CVI (23). The CVI was calculated on a 4-point scale, with one denoting no relevance, two denoting low relevance, three denoting great relevance, and four denoting extremely high relevance. Each expert judged the extent to which each item was related to the scale.

##### 2.5.2.3. Structural validity

To assess the structural validity of the Chinese version of the Geriatric Anxiety Scale-Long-Term Care scale, EFA and CFA techniques were used. Two samples were created by randomly dividing the data. EFA was performed on Sample 1 (*n* = 209), and the KMO (Kaiser-Meyer-Olkin) statistic (24) and Bartlett’s test of sphericity (25) were used for dimensionality. Based on the results of exploratory factor analysis, a validated factor analysis was performed on sample 2 (*n* = 199). Using squared degrees of freedom (χ^2^/df), comparative fit index (CFI) (26), Tucker Lewis index (TLI) (27), standardized root mean square residual (SRMR), and root mean square error of approximation (RMSEA) to assess model fit. An acceptable model should have *x*^2^/df < 3, RMSEA and SRMR <0.08, and CFI and TLI > 0.9 (21, 22).

##### 2.5.2.4. Linear regression analysis

The Pearson correlation analysis method was employed to screen the independent variables and investigate their relationship with anxiety in terms of demographic variables. Variables showing significant correlations were selected as independent variables, with anxiety being the dependent variable. Stepwise selection method was utilized for conducting multiple linear regression analysis. To assess the effectiveness of the model, the adjusted R-squared is utilized to measure the goodness of fit, and the significance of the entire regression model is tested using the *F*-value and its corresponding *p*-value (19). The reliability of the model is evaluated through significance tests conducted on the regression coefficients, involving the calculation of standard errors, *t*-values, and *p*-values. This analysis determines the significance of the independent variables’ impact on the dependent variable (19). The prediction factors (28) are examined by considering the direction and magnitude of the regression coefficients, which indicate the direction and strength of the relationship between the independent and dependent variables. Collinearity diagnosis and the plotting of a residual scatter plots are employed to conduct hypothesis testing for the model assumptions.

## 3. Results

### 3.1. Descriptive statistics

The demographic characteristics of the participants are detailed in Tables 1, 2. Scale Cultural Adjustment study participants (Table 1) were males (48.6%, *n* = 194) and females (51.4%, *n* = 205) with a mean age of 68.64 ± 5.887 years. Participants in the study of influences affecting anxiety in the long-term care population (Table 2) were males (48.1%, *n* = 180) and females (51.9%, *n* = 194) with a mean age of 68.85 ± 6.057 years.

**Table 1:** Demographic characteristics.

Characteristics	Total (*N* = 399) *N* (%)/M ± SD
Age(years)	68.64 ± 5.887
Gender	
Male	194 (48.6%)
Female	205 (51.4%)
Education level	
Primary school or below	316 (79.2%)
Junior high school	56 (14.0%)
High school or technical secondary school	13 (3.3%)
College degree or above	14 (3.5%)
Smoke	
Yes	177 (44.4%)
No	222 (55.6%)
Drink	
Yes	104 (26.1%)
No	295 (73.9%)

SD, standard deviation.

**Table 2:** Demographic characteristics.

Characteristics	Total (*N* = 374) *N* (%)/M ± SD
Age(years)	68.85 ± 6.057
Gender	
Male	180 (48.1%)
Female	194 (51.9%)
Education level	
Primary school or below	305 (81.6%)
Junior high school	45 (12.0%)
High school or technical secondary school	10 (2.7%)
College degree or above	14 (3.7%)
Marital status	
Unmarried	71 (19.0%)
Married	55 (14.7%)
Divorced/widowed	248 (66.3%)
Smoke	
Yes	95 (25.4%)
No	279 (74.6%)
Drink	
Yes	167 (44.7%)
No	207 (55.3%)
Self-rated health	
Very good	11 (2.9%)
Good	12 (3.2%)
Fair	136 (36.4%)
Poor	147 (39.3%)
Very poor	68 (18.2%)
Life satisfaction	
Not at all satisfied	18 (4.8%)
Not very satisfied	37 (9.9%)
Somewhat satisfied	198 (52.9%)
Very satisfied	109 (29.1%)
Completely satisfied	12 (3.2%)
Participation activities	
Yes	139 (37.2%)
No	235 (62.8%)
Communication with children	
Yes	316 (84.5%)
No	58 (15.5%)
Retirement	
Yes	204 (54.5%)
No	170 (45.5%)

### 3.2. Item analysis

The item analyzes of the Geriatric Anxiety Scale-Long-Term Care are shown in Table 3. The critical ratio CR > 3.000 indicates that the scale has good discriminative validity of the entries, and the CR values of the 10 entries of the scale ranged from 7.351 ~ 29.173, which suggests that the scale has good discriminant validity. The correlation coefficients between the entries and the total score of the scale ranged from 0.348 ~ 0.743, and the differences were statistically significant. In addition, the internal consistency of the entire scale was not significantly improved by deleting items.

**Table 3:** Reliability analysis.

Items	Yes	Mean	SD	Critical ratio	Correlation coefficient between item and total score	Cronbach’s Alpha if item deleted
1	93 (44.5%)	0.44	0.498	29.173	0.743	0.768
2	67 (32.1%)	0.32	0.468	14.353	0.652	0.782
3	82 (39.2%)	0.39	0.489	13.915	0.599	0.789
4	88 (42.1%)	0.42	0.495	14.976	0.594	0.790
5	89 (42.6%)	0.43	0.496	15.905	0.618	0.787
6	99 (47.4%)	0.47	0.501	20.465	0.694	0.776
7	100 (47.8%)	0.48	0.501	7.351	0.348	0.821
8	107 (51.2%)	0.51	0.501	21.565	0.654	0.782
9	103 (49.3%)	0.49	0.501	12.414	0.559	0.795
10	63 (30.1%)	0.30	0.460	12.659	0.573	0.791

### 3.3. Reliability analysis

The Chinese version of the Geriatric Anxiety Scale-Long-Term Care scale consists of 10 items. The KR-20 reliability coefficient is 0.81, Cronbach’s alpha value is 0.81, and the split-half reliability is 0.80. The values of the correlation coefficients are all above 0.3. All of these findings indicate that the Chinese version of the Geriatric Anxiety Scale-Long-Term Care scale demonstrates good reliability.

### 3.4. Exploratory factor analysis and model comparison

The Bartlett’s test for sphericity for exploratory factor analysis (EFA) was significant (*x*^2^ = 540.831, *p* < 0.001), with a KMO index of 0.890. The results of the EFA showed that the factor loadings of the items ranged from 0.329 to 0.783 (Table 4).

**Table 4:** Factor loadings for the Chinese version of the Geriatric Anxiety Scale-Long-Term Care.

	Items	Factor
1	I was irritable or grumpy	0.783
2	I felt detached or isolated from others	0.648
3	I felt like I was in a daze or foggy-headed	0.678
4	I had a hard time sitting still	0.505
5	I could not control my worry	0.595
6	I felt restless, keyed up, or on edge	0.735
7	I felt overly tired	0.329
8	My muscles were tense or tight	0.696
9	I felt like I had no control over my life	0.609
10	I felt like something terrible was going to happen to me	0.639

Based on the factor distributions of EFA, a CFA model was constructed using MPLUS, and the model was fitted and analyzed. The one-factor model fit index (*x*^2^/df = 1.148, CFI = 0.992, TLI = 0.990, RMSEA = 0.066 [90% CI: 0.000 ~ 0.061], SRMR = 0.065). The results indicated a statistically acceptable fit for the one-factor model.

### 3.5. Content validity

Experts were invited to assess the content validity of the Chinese version of the Geriatric Anxiety Scale-Long-Term Care scale (29). A total of seven experts were invited to conduct the assessment, and the results of the content validity analysis showed that the Geriatric Anxiety Scale-Long-Term Care has good content validity, with an I-CVI of 0.857 ~ 1.000 and an S-CVI/Ave of 0.971.

### 3.6. Multivariate regression linear analysis

**Figure 1:**
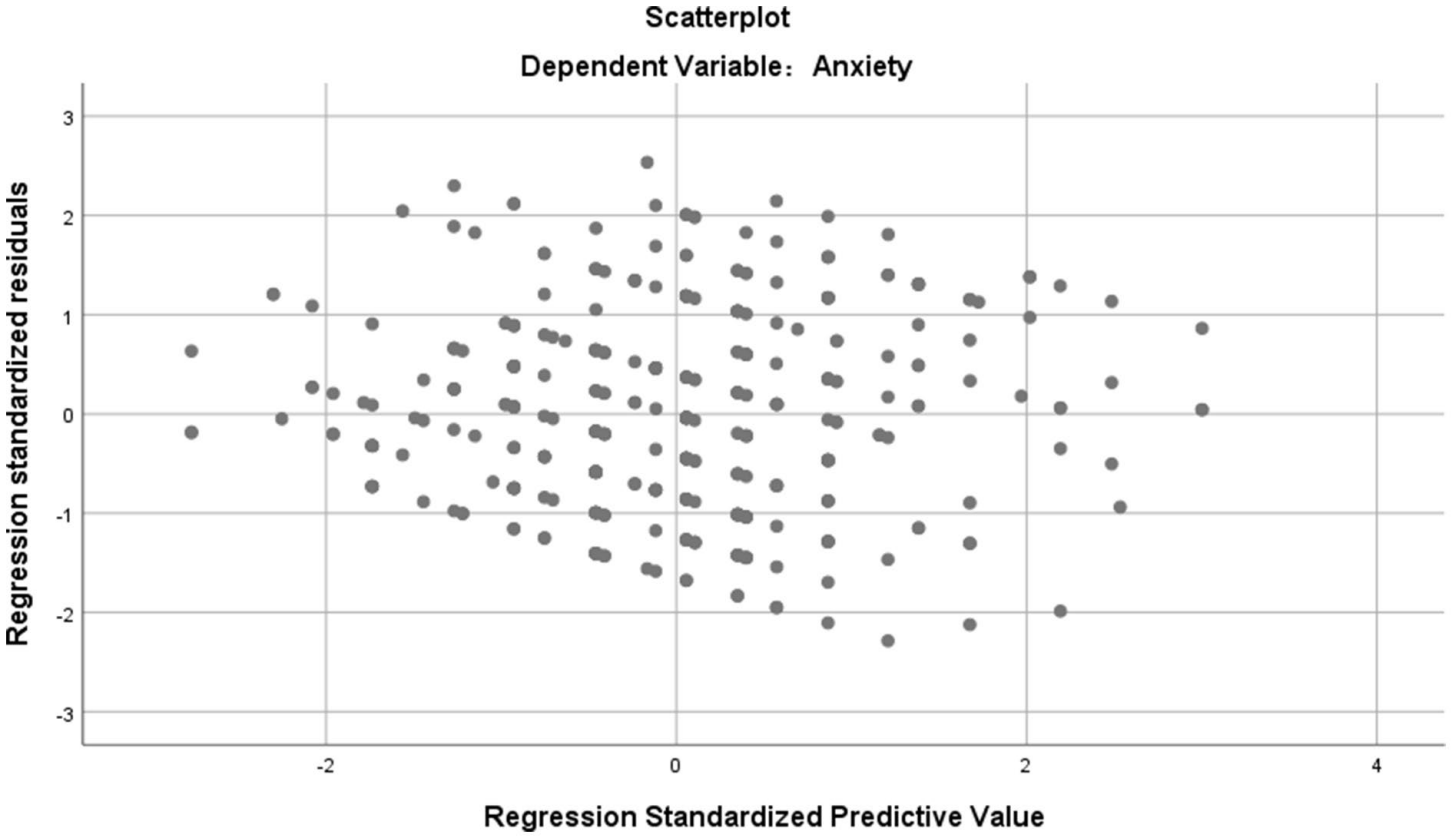
Residual scatterplot.

The results of the correlation analysis are presented in Table 5, revealing significant correlations between anxiety and variables such as age, gender, self-rated health, drinking, activity participation, and life satisfaction (*p* < 0.05). These variables were selected as independent variables and subjected to multiple linear regression analysis. The findings indicated an adjusted R-squared value of 0.211, *F* = 26.003, *p* < 0.001. The regression equation demonstrated statistical significance, suggesting a good fit between the independent variables and the outcome variable. Additionally, the variance inflation factor (VIF) values (Table 6) were all below 5, indicating no substantial multicollinearity issues. Furthermore, the residual plots exhibited no discernible patterns, indicating the absence of significant violations of regression assumptions (Figure 1). Overall, the model testing results were deemed satisfactory. The results of multiple regression analysis showed that the total score of the anxiety scale of the older adults in relation to gender (*β* = 0.190, 95% CI: 0.540 ~ 1.546, *p* < 0.001), life satisfaction (*β* = −0.315, 95% CI:−1.355 ~ −0.734, *p* < 0.001), self-rated health (*β* = 0.220, 95% CI: 0.379 ~ 0.953, *p* < 0.001), and activity participation (*β* = −0.106, 95%CI: −1.122 ~ −0.084, *p* < 0.05) were significantly correlated (Table 6).

**Table 5:** Correlation analysis between total anxiety score and demographic variables.

Variables	Correlation	*p*	Variables	Correlation	*p*
Age	−0.110	0.034	Communication with children	−0.072	0.167
Gender	0.151	0.003	Participation activities	−0.109	0.034
Education level	−0.100	0.053	Retirement	−0.047	0.367
Self-rated health	0.287	0.000	Life satisfaction	−0.370	0.000
Drink	−0.127	0.014	Marital status	0.045	0.384
Smoke	−0.012	0.820			

**Table 6:** Effect of sociodemographic factors on anxiety total scores: linear regression analysis.

Model	Beta	*t*	*p*	95% CI	VIF
Constant		3.655	0.000	1.629 ~ 5.422	1.073
Gender	0.190	4.075	0.000	0.540 ~ 1.546	1.020
Life satisfaction	−0.315	−6.611	0.000	−1.355 ~ −0.734	1.105
Self-rated health	0.220	4.560	0.000	0.379 ~ 0.953	1.026
Participation activities	−0.106	−2.284	0.023	−1.122 ~ −0.084	0.622

## 4. Discussion

As the aging population increases, most older adults individuals face health challenges and functional decline, which may require long-term care support and increase the likelihood of experiencing anxiety symptoms. However, there is currently no specific measurement tool for anxiety symptoms in older adults individuals requiring long-term care in China. The Generalized Anxiety Scale (GAS) is commonly used in China to measure anxiety, but its lack of population specificity reduces sensitivity. Professor Segal (2) has developed the Geriatric Anxiety Scale-Long-Term Care scale specifically for long-term care populations based on their unique needs and characteristics, using a “yes” or “no” response mechanism to make answering easier. In order to fill the gap in the lack of anxiety assessment tools for long-term care populations in China, this study aims to translate and ensure the reliability of the Geriatric Anxiety Scale-Long-Term Care scale and introduce it to China. Additionally, this study explores the factors influencing anxiety in long-term care populations. The results of this study indicate that the Chinese version of the Geriatric Anxiety Scale-Long-Term Care is an effective and reliable tool for measuring anxiety symptoms in older adults individuals requiring long-term care in China.

According to Brislin’s principles of translation, the Chinese version of the Geriatric Anxiety Scale-Long-Term Care was completed in this study, and the translation was adjusted by the research team according to the relevant guidelines and Chinese expression habits to ensure that the Chinese scale was fully equivalent to the original scale. In the pre-test, the Geriatric Anxiety Scale-Long-Term Care was administered to 20 older adults in the long-term care population, and it was found that the scale structure and semantics were simple and easy to understand. In addition, the critical ratios (CR) of all items of the Chinese version of the Geriatric Anxiety Scale-Long-Term Care scale were > 3.000, and deletion of any item did not improve the internal consistency of the whole scale, indicating strong discriminant validity of the scale. The correlation coefficients between the items and the total score ranged from 0.348 ~ 0.743. The Cronbach’s alpha value of the translated scale was 0.81, which was slightly higher than that of the original scale (2), and the split-half reliability was 0.80. Therefore, the Chinese version of the Geriatric Anxiety Scale-Long-Term Care scale has sufficient reliability among long-term care residents.

The reliability of the Geriatric Anxiety Scale-Long-Term Care was evaluated in this study by assessing its content validity and structural validity. The content validity was determined by calculating the Item-Content Validity Index (ICVI), which was found to be 0.8571, and the Scale-Content Validity Index (S-CVI), which was 0.971. These values were higher than the reference value for content validity (30), indicating that the Geriatric Anxiety Scale-Long-Term Care has strong content validity. Exploratory factor analysis revealed that one factor accounted for 40.124% of the total data variance, and the factor loading of each item was greater than 0.3 (31, 32), indicating good structural validity. Furthermore, we confirmed the one-factor model of the Chinese version of the Geriatric Anxiety Scale-Long-Term Care with good overall model indicators. Based on these findings, we conclude that the Chinese version of the Geriatric Anxiety Scale-Long-Term Care is a valid tool for assessing anxiety in the long-term care facility resident population.

This study found that gender influences anxiety in the long-term care population, which is consistent with previous study (33–37) and may be related to the fact that women are more susceptible to negative influences (33). Additionally anxiety in the long-term care population was influenced by self-assessed health status, which is consistent with previous study (37–40) and may be due to the fact that higher self-assessed health is associated with more positive emotions. When individuals have higher self-health reports of their health status, it suggests that they are more confident in their health and are more likely to adopt a positive attitude towards life (41), which triggers the development of anxiety when people are concerned about the potential consequences of poor health (42). In addition, this study found that anxiety in the long-term care population was influenced by life satisfaction, which is consistent with previous study (43–45). It is not difficult to understand that life satisfaction reflects older adults’ overall satisfaction with all aspects of their lives, and when they experience dissatisfaction or face difficulties, they may be more prone to adverse emotions. The results of this study show that participation in activities affects anxiety in the long-term care population, which is consistent with previous study (46–48). This may be related to the fact that engaging in activities reduces loneliness among older adults. Severe feelings of loneliness are often associated with negative emotional states and adverse psychological consequences that can lead to various physical and mental health issues, including anxiety (49). In contrast, participation in activities can provide older adults with a sense of presence and fulfillment and reduce feelings of loneliness.

## 5. Limitations

This study has some limitations that should be noted. Firstly, a retest reliability test was not conducted. Secondly, although the sample size was sufficient for the study, it was not a multi-province study, and the relatively narrow selection process may limit the generalizability of the findings. Therefore, future studies should aim to expand the scope and sample to include a more diverse population to improve the generalizability of the results.

## 6. Conclusion

This study employed a rigorous process for the translation, back-translation, cross-cultural adaptation, pre-experimentation, reliability, and validity testing of the Chinese version of the Geriatric Anxiety Scale-Long-Term Care. The Geriatric Anxiety Scale-Long-Term Care was successfully introduced into China with good validity and reliability. It is an appropriate measurement tool to quickly assess the anxiety levels of Chinese older adults in need of long-term care and to provide a basis and prerequisite for researchers to develop educational programs and interventions in the context of geriatric caregiver shortage and population aging.

## Data availability statement

The datasets presented in this article are not readily available because the datasets generated and/or analyzed during the current study are not publicly available to preserve anonymity of the respondents but are available from the corresponding author on reasonable request. Requests to access the datasets should be directed to 1377533362@qq.com.

## Ethics statement

The studies involving humans were approved by Jinzhou Medical University (Ethics approval number: JZMULL2022095). The studies were conducted in accordance with the local legislation and institutional requirements. The participants provided their written informed consent to participate in this study.

## Author contributions

FF: Writing – original draft. QC: Writing – review & editing. CZ: Writing – review & editing. HZ: Writing – review & editing.
